# Implementation of an In-House Platform for Rapid Screening of SARS-CoV-2 Genome Variations

**DOI:** 10.34172/aim.2023.12

**Published:** 2023-02-01

**Authors:** Farzane Zare Ashrafi, Marzieh Mohseni, Maryam Beheshtian, Zohreh Fattahi, Fatemeh Ghodratpour, Fatemeh Keshavarzi, Hanieh Behravan, Marzieh Kalhor, Khadijeh Jalalvand, Maryam Azad, Mahdieh Koshki, Ali Jafarpour, Azam Ghaziasadi, Alireza Abdollahi, Seyed Jalal Kiani, Angila Ataei-Pirkooh, Iman Rezaei Azhar, Farah Bokharaei-Salim, Mohammad Reza Haghshenas, Farhang Babamahmoodi, Zakiye Mokhames, Alireza Soleimani, Masood Ziaee, Davod Javanmard, Shokouh Ghafari, Akram Ezani, Alireza Ansari Moghaddam, Fariba Shahraki-Sanavi, Seyed Mohammad Hashemi Shahri, Azarakhsh Azaran, Farid Yousefi, Afagh Moattari, Mohsen Moghadami, Hamed Fakhim, Behrooz Ataei, Elahe Nasri, Vahdat Poortahmasebi, Mojtaba Varshochi, Ali Mojtahedi, Farid Jalilian, Mohammad Khazeni, Abdolvahab Moradi, Alijan Tabarraei, Ahmad Piroozmand, Yousef Yahyapour, Masoumeh Bayani, Amir Aboofazeli, Parsa Ghafari, Fariba Keramat, Mahsa Tavakoli, Tahmineh Jalali, Mohammad Hassan Pouriayevali, Mostafa Salehi-Vaziri, Hamid Reza Khorram Khorshid, Reza Najafipour, Reza Malekzadeh, Kimia Kahrizi, Seyed Mohammad Jazayeri, Hossein Najmabadi

**Affiliations:** ^1^Genetics Research Center, University of Social Welfare and Rehabilitation Sciences, Tehran, Iran; ^2^Kariminejad-Najmabadi Pathology & Genetics Center, Tehran, Iran; ^3^Research Center for Clinical Virology, Tehran University of Medical Sciences, Tehran, Iran; ^4^Gerash Amir-al-Momenin Medical and Educational Center, Gerash University of Medical Sciences, Gerash, Iran; ^5^Department of Pathology, School of Medicine, Imam Khomeini Hospital, Tehran University of Medical Sciences, Iran; ^6^Department of Virology, School of Medicine, Iran University of Medical Sciences, Tehran, Iran; ^7^Department of Medical Microbiology, Antimicrobial Resistance Research Center, Communicable Diseases Institute, Faculty of Medicine, Mazandaran University of Medical Sciences, Sari, Iran; ^8^Department of Molecular Diagnostic, Emam Ali Educational and Therapeutic Center, Alborz University of Medical Sciences, Karaj, Iran; ^9^Department of Infectious Diseases, Imam Ali hospital, Alborz University of Medical Sciences, Karaj, Iran; ^10^Infectious Diseases Research Center, Birjand University of Medical Sciences, Birjand, Iran; ^11^Qazvin Deputy of Treatment Reference Laboratory, Qazvin University of Medical Sciences, Qazvin, Iran; ^12^Health Promotion Research Center, Zahedan University of Medical Science, Zahedan, Iran; ^13^Infection Disease and Tropical Medicine Research Center, Zahedan University of Medical Science, Zahedan, Iran; ^14^Department of Medical Virology, School of Medicine, Ahvaz Jundishapur University of Medical Sciences, Ahvaz, Iran; ^15^Department of Infectious Diseases, School of Medicine, Razi Hospital, Ahvaz Jundishapur University of Medical Sciences, Ahvaz, Iran; ^16^Department of Virology, School of Medicine, Shiraz University of Medical Sciences, Shiraz, Iran; ^17^Health policy research center, Shiraz University of medical sciences, Shiraz, Iran; ^18^Infectious Diseases and Tropical Medicine Research Center, Isfahan University of Medical Sciences, Isfahan, Iran; ^19^Department of Bacteriology and Virology, Faculty of Medicine, Tabriz University of Medical Sciences, Tabriz, Iran; ^20^Infectious and Tropical Disease Research Center, Tabriz University of Medical Science, Tabriz, Iran; ^21^Microbiology Department, School of Medicine, Guilan University of Medical Sciences, Rasht, Iran; ^22^Department of Medical Virology, Faculty of Medicine, Hamadan University of Medical sciences, Hamadan, Iran; ^23^Booali lab, Molecular & Virology Diagnostic Section, Qom, Iran; ^24^Golestan University of Medical Sciences, Gorgan, Iran; ^25^Department of Microbiology, School of Medicine, Kashan University of Medical Sciences, Kashan, Iran; ^26^Infectious Diseases and Tropical Medicine Research Center, Babol University of Medical Sciences, Babol, Iran; ^27^Brucellosis Research Center, Hamedan University of Medical Science, Hamadan, Iran; ^28^COVID-19 National Reference Laboratory, Pasteur Institute of Iran, Tehran, Iran; ^29^Department of Arboviruses and Viral Hemorrhagic Fevers (National Reference Laboratory), Pasteur Institute of Iran, Tehran, Iran; ^30^Cell and Molecular Research Center, Qazvin University of Medical Sciences, Qazvin, Iran; ^31^Digestive Disease Research Institute, Shariati Hospital, Tehran University of Medical Sciences, Tehran, Iran

**Keywords:** COVID-19, Nested RT-PCR, Sanger sequencing, SARS-CoV-2 variants, Spike gene

## Abstract

**Background:**

Global real-time monitoring of SARS-CoV-2 variants is crucial to controlling the COVID-19 outbreak. The purpose of this study was to set up a Sanger-based platform for massive SARS-CoV-2 variant tracking in laboratories in low-resource settings.

**Methods:**

We used nested RT-PCR assay, Sanger sequencing and lineage assignment for 930-bp of the SARS-CoV-2 spike gene, which harbors specific variants of concern (VOCs) mutations. We set up our platform by comparing its results with whole genome sequencing (WGS) data on 137 SARS-CoV-2 positive samples. Then, we applied it on 1028 samples from March-September 2021.

**Results:**

In total, 125 out of 137 samples showed 91.24% concordance in mutation detection. In lineage assignment, 123 out of 137 samples demonstrated 89.78% concordance, 65 of which were assigned as VOCs and showed 100% concordance. Of 1028 samples screened by our in-house method, 78 distinct mutations were detected. The most common mutations were: S:D614G (21.91%), S:P681R (12.19%), S:L452R (12.15%), S:T478K (12.15%), S:N501Y (8.91%), S:A570D (8.89%), S:P681H (8.89%), S:T716I (8.74%), S:L699I (3.50%) and S:S477N (0.28%). Of 1028 samples, 980 were attributed as VOCs, which include the Delta (B.1.617.2) and Alpha (B.1.1.7) variants.

**Conclusion:**

Our proposed in-house Sanger-based assay for SARS-CoV-2 lineage assignment is an accessible strategy in countries with poor infrastructure facilities. It can be applied in the rapid tracking of SARS-CoV-2 VOCs in the SARS-CoV-2 pandemic.

## Introduction

 A novel coronavirus, entitled severe acute respiratory syndrome coronavirus 2 (SARS-CoV-2), emerged at the end of 2019 in the city of Wuhan, China, and caused the coronavirus disease 2019 (COVID-19) pandemic, leading to more than 552504629 infections and six million deaths around the world as of July 11, 2022.^1-3^

 The SARS-CoV-2 is a spherical enveloped *betacoronavirus* with non-segmented, positive single-stranded RNA with a genome size of nearly 30 kb,^4^ showing 79% genomic sequence similarity with SARS-CoV and 50% with MERS-CoV; the two known coronaviruses identified in 2002 and 2012, respectively.^2,4,5^ The genome structure of SARS-CoV-2 translates to nonstructural proteins (NSP1–16), structural proteins of the virus including S (spike), E (envelope), M (membrane), N (nucleocapsid) and several accessory proteins.^5^ The spike (S) proteins are homotrimeric glycoproteins with a total length of 1273 amino acids that are encoded by a 3783bp S gene and involved in binding to angiotensin-converting enzyme 2 (ACE2) receptors and virus entry into host cells.^2,6,7^ S proteins are constituted of the S1 subunit which has a 211-amino acid region called receptor-binding domain (RBD) that has a crucial role in viral entry by recognition and attachment to the host ACE2 receptor; the S2 subunit with heptad repeat regions and the fusion peptide is involved in viral membrane fusion.^2,5,7^ Mutations in the spike gene can result in the increase of virus transmissibility, infectivity and immune escape and make it a major target for research studies, diagnostic and therapeutic strategies and vaccine development.^8-10^

 Whole genome sequencing (WGS) of SARS-CoV-2 by next-generation sequencing (NGS) has characterized the complete genome sequence of the virus since the beginning of the pandemic, allowing detection of all possible lineages and variants of the virus which can be used for designing various diagnostic methods, drugs and vaccines development.^11,12^ As of July 12, 2022, approximately 11 642 776 complete SARS-CoV-2 genome sequences have been deposited in the Global Initiative on Sharing All Influenza Data (GISAID) EpiCoV^TM^ public database.^13^ Considering the continuous evolution of SARS-CoV-2 and the emergence of several variants with specific phenotypic features which can threaten global public health, the World Health Organization (WHO) designated “variants of interest” (VOIs) or “variants of concern” (VOCs) for prioritization of worldwide monitoring and research studies.^14,15^ Variants accompanied by expanded transmissibility, disease severity and decline in efficacy of vaccines or therapeutics are classified as VOCs and need strict screening.^8^ Overall, five VOCs have been declared by the WHO, namely, Alpha (B.1.1.7), Beta (B.1.351), Gamma (P.1), Delta (B.1.617.2) and Omicron (B.1.1.529) which were respectively first identified in the United Kingdom, South Africa, Brazil, India and South Africa.^8,16,17^

 Despite the fact that Iran was one of the first and most affected countries facing the SARS-CoV-2 rapid expansion, there was no large-scale SARS-CoV-2 genome sequencing data available for monitoring the circulating SARS-CoV-2 variants throughout the country in the early days of the pandemic.^18,19^ A considerable effort by the Genetics Research Center (GRC) of the University of Social Welfare and Rehabilitation Sciences (USWR) subjected 369 SARS-CoV-2 samples from different regions of Iran to WGS from March 2020 to May 2021.^18,19^

 Although WGS is the commonly used platform^12^ applied for SARS-CoV-2 genomic surveillance, several factors including long turnaround time, expensiveness, and the need for appropriate sequencing facilities with trained staff are not routinely accessible in all laboratories. Therefore, the establishment of an alternative method is of paramount importance.^11,20^ Considering the critical functions of RBD in viral attachment to the receptor cells, Sanger sequencing of RBD in the S gene can be used as one of the best alternative approaches to identifying most of the known variants of SARS-CoV-2.^21,22^

 We developed an in-house platform comprising a nested RT-PCR assay followed by Sanger sequencing to target characteristic mutations in the crucial region of the SARS-CoV-2 Spike gene. This method can be applied routinely for rapid scanning of SARS-CoV-2 positive samples in laboratories with basic Sanger sequencing facilities.

## Materials and Methods

###  Patient Recruitment

 We obtained 1028 SARS-CoV-2 positive RNA samples from all over the country between March 2021 and September 2021 from private laboratories, the COVID-19 laboratory network and the Iranian Network for Research in Viral Diseases (INRVD). These centers have performed viral RNA extractions from respiratory samples (Naso/Oro-pharyngeal swabs) using standard protocols and SARS-CoV-2 infection confirmation via real-time RT-PCR assays.

###  Primer Selection

 Our study used the RT-Nested primers to target the regions between the nucleotide positions 22886 and 23796 of the Wuhan (Wu-1) reference genome (GenBank accession: NC_045512.2) of the SARS-CoV-2 Spike gene, partly, covering characteristic mutations of VOCs as shown in Table 1. The primers used in the first and second rounds of RT-Nested PCR were^23^: Nested1-F: 5’-TTACAGGCTGCGTTATAGCTTGG-3’, Nested1-R: 5’-TGCTGCATTCAGTTGAATCACC-3’, Nested2-F: 5’-GCTTGGAATTCTAACAATCTTG-3’, Nested2-R: 5’-TCACCACAAATGTACATTGTAC-3’.

**Table 1:** Lineage Assignment Based on Significant Mutations in Our Study

**Pango Lineage**	**B.1.1.7**	**B.1.351**	**P.1**	**B.1.617.2**
**WHO Label**	**Alpha**	**Beta**	**Gamma**	**Delta**
L452R				+
T478K				+
E484K		+	+	
N501Y	+	+	+	
A570D	+			
D614G	+	+	+	+
H655Y			+	
P681H	+			
P681R				+
A701V		+		
T716I	+			

###  Nested RT-PCR Assay and Sanger Sequencing

**Figure 1:**
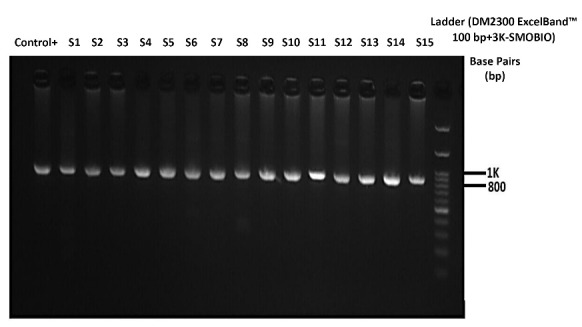
Agarose-gel (2%) Electrophoresis of the 931bp Products of RT-Nested PCR

 Nested RT-PCR assay was performed with SMOBIO [RQ2110] ExcelRT^TM^ One-Step RT-qPCR Kit and the Nested RT- PCR conditions described in Table 2. The 931-bp PCR products were analyzed by 2% agarose gel electrophoresis (Figure 1). After purification with ExoSAP-IT (Affymetrix), sequencing reactions were performed with the BigDyeTM Terminator v3.1 Cycle Sequencing Kit by Applied Biosystems Genetic Analyzer, following the manufacturer’s instructions.

**Table 2:** Nested RT-PCR Conditions

**Nested 1**						
RT-PCR set-up	**Reagent**	**Volume (µL)**	Cycling conditions	**Temperature**	**Time**	
	2X One-Step Master Mix^*^	17		60°C	1 min	Decrease 0.5 C°/s
	Water	2		50°C	30 min	
	Forward primer	0.25		94°C	15 s	40 cycles
	Reverse primer	0.25		55°C	15 s	
	One-Step RT Enzyme Mix^*^	0.5		72°C	1 min	
	Template (RNA)	5		72°C	7 min	
	Total	25		4°C	Hold	
**Nested 2**						
RT-PCR set-up	**Reagent**	**Volume (µL)**	Cycling conditions	**Temperature**	**Time**	
	Master Mix	18		94°C	3 min	
	Forward primer	0.5		94°C	15 s	30 cycles
	Reverse primer	0.5		55°C	15 s	
	SuperTaq DNA Polymerase 500 U (5U/µL) 100 µL	0.2		72°C	1 min	
	Template (1^st^ round PCR Product)	3		72°C	7 min	
	Total	22.2		4°C	Hold	

[RQ2110] ExcelRT^TM^ One-Step RT-qPCR Kit (TaqMan, ROX)-SMOBIO.

###  Sanger Sequencing-based Mutation Analysis 

 The general protocol in our in-house platform was as follows:

1. Manually mutation analysis of the electropherograms, using CodonCode Aligner v.9.0.1 (Figure 2).
  **Figure 2:**
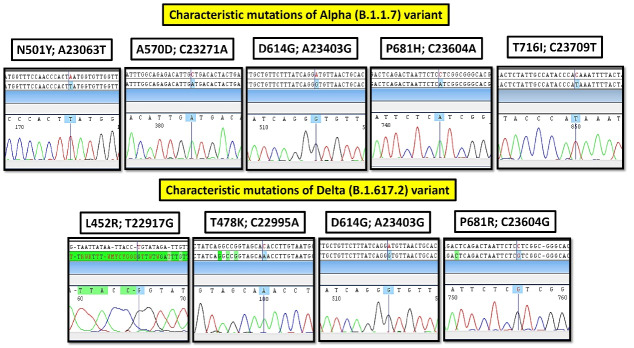
The Sequencing Electropherograms of Characteristic Mutations of Alpha (B.1.1.7) and Delta (B.1.617.2) Variants
2. The probable lineage assignment of each sample according to the VOCs key mutations shown in Table 1.^8,10,14,15,24^

Lineage assignment for samples that did not harbor these significant sets of mutations was performed with the help of https://outbreak.info/ and several studies in the literature (Table 1).^24,25^

 Before applying this proposed method for monitoring VOCs on 1028 SARS-CoV-2 positive samples, we checked the concordance of Sanger and NGS results by blind comparison of these two platforms on 137 samples. It should be noted that most of these 137 samples were the cases investigated in the two previous studies of our group and the others were among the samples collected for this project. The details of the WGS method and lineage assignment of samples have been mentioned in our two previous studies.^18,19^

## Results

 A comparison of Sanger and WGS results in 137 of our samples showed (125/137) 91.24% concordance in mutation detection and (123/137) 89.78% concordance in lineage assignment. It should be noted that of these 137 samples, 65 were attributed as VOCs and 72 were attributed as non-VOCs. Mutation detection and lineage assignment in VOCs had 87% and 100% concordance, respectively.

 In total, 1028 SARS-CoV-2 samples were studied from 10 different provinces of Iran between March and September 2021. The number of samples monitored from each province in this cohort is as follows: Tehran 479 (46.60%),Alborz 212 (20.62%), Sistan and Baluchestan 111 (10.80%), Semnan 80 (7.78%), Gilan 39 (3.79%), Fars 34 (3.31%), Markazi 21 (2.04%), Isfahan 19 (1.85%), Mazandaran 18 (1.75%) and Golestan 15 (1.46%). Also, the number of sequenced samples per month was: 21 March-20 April 2021(79), 21 April-21 May 2021(136), 22 May-21 June 2021(160), 22 June-22 July 2021(235), 23 July-22 August 2021(191), 23 August-22 September 2021(227) (Supplementary File 1).

**Figure 3:**
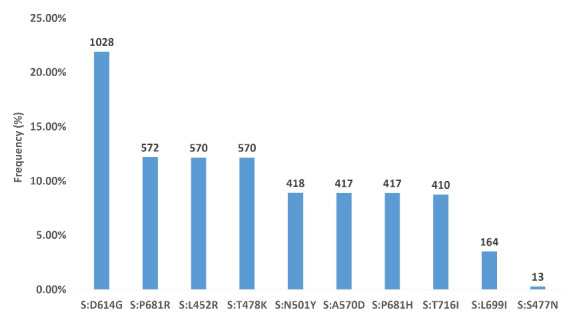
Top 10 Mutations Detected in 1028 Samples Studied

 We detected 78 distinct mutations in the regions between the nucleotide positions 22886 and 23 796 of the spike gene; Figure 3 shows the top ten mutations detected in 1028 samples of the cohort. The most frequent mutation observed in our study was S:D614G substitution, constituting 21.91% of all mutations. Other common spike mutations were: S:P681R (12.19%), S:L452R (12.15%), S:T478K (12.15%), S:N501Y (8.91%), S:A570D (8.89%), S:P681H (8.89%), S:T716I (8.74%), S:L699I (3.50%) and S:S477N (0.28%).

 Among the 1028 sequenced samples, 570 samples were assigned to Delta (B.1.617.2), 410 samples to Alpha (B.1.1.7), 22 samples to (B.1), ten samples to (B.1.160), and three samples to (B.1.617.1 or B.1.617.3). Thirteen samples failed to assign a distinct lineage because the presence of only a small number of spike mutations was not enough for conclusive lineage assignment, and these mutations were seen in many Pango lineages.

**Figure 4:**
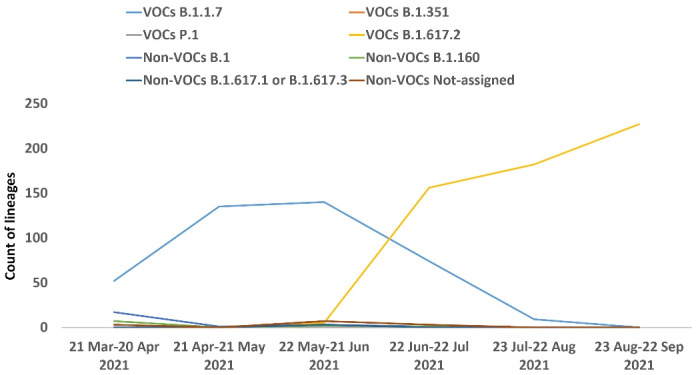
Trend of Lineages Identified by Month in Samples Studied

 The most frequent lineage observed in our cohort was Delta (B.1.617.2), and overall, the Alpha (B.1.1.7) and Delta (B.1.617.2) variants were the VOCs distinguished in our cohort, and no samples contained the Beta (B.1.351) and Gamma (P.1) variants. Figure 4 shows the lineages identified each month in our cohort (Supplementary File 1).

## Discussion

 Considering the role of the spike protein in binding to ACE2 receptors and entry to the host cells and its importance in developing vaccines and antibody-based therapeutics strategies, scanning of SARS-CoV-2 spike gene mutations is important.^9,21^ Although NGS platforms would be ideal for whole-genome surveillance of SARS-CoV-2 variants and comprehensive analysis of the spike gene region, massive sequencing of SARS-CoV-2 samples by these platforms is still impossible in many developing and underdeveloped countries due to factors such as high cost, limited supplies and infra-structure and the need for skilled staff for WGS.^12,20^ Therefore, establishing alternative platforms for massive monitoring of SARS-CoV-2 VOCs can help overcome the challenges. So, we proposed an alternative sanger-based platform for lineage assignment of SARS-CoV-2 samples by analyzing characteristic mutations in a distinct part of the spike gene. Sanger sequencing with 99.99% accuracy is widely accessible in most laboratories.^26^ It can sequence fragments with relatively low cost, rapid turnaround time, and conditions of low viral loads.^12,27^ Despite all these advantages, the length of amplicons is limited in Sanger sequencing (often less than 1000 bp). It is practically impossible to sequence long fragments like the whole genome of SARS-CoV-2 or the full spike as one amplicon.^12,26,27^ So, we had to select a more critical part of the spike for lineage assignment just by one amplicon. Our selected 930 bp region included characteristic mutations of VOCs in the period when we set up this protocol.

 According to our study, the selected region in Spike was successfully amplified and sequenced, and the probable lineages were assigned. Among all 137 compared NGS and Sanger data, 73 samples (53.28%) were compatible and included B.1.1.7 and B.1 lineages; 50 samples (36.50%) were partially compatible, consisting of B.1.1, B.1.1 with (S:D138Y, S:S477N, S:D614G)/B.1.1.413 (v.3.1.11), B.1.1.10, B.1.1.317, B.1.1.326, B.1.36, B.1.36.10, B.1.36.7, B.1.456 and B.1.468 lineages, and also 14 samples (10.22%) were not assigned (Supplementary File 2). The use of the terms “Compatible”, “Partially Compatible”, and “Incompatible” in lineage assignment for comparing the results of the two platforms well covered the classification of samples. The term “Compatible” includes samples with 100% similarity and “Incompatible” refers to completely different samples. The term “Partially Compatible” is used for samples that differed in ascribing the exact sub-lineage but both descended from a similar either lineage, A or B. Also, the use of the word “Not assigned” in determining the lineages included samples that either did not have mutations in the spike or the mutations detected in the spike corresponded to many Pango lineages.

 The distribution of 1028 investigated samples is related to just ten Iran provinces, of which about 82% are located in the northern parts of the country, 14.11% in the southern regions and 3.89% in the central parts; it should be noted that the following statistics might mostly reflect the dominant lineages in these areas. Our study showed that 980 out of 1028 samples were designated as VOCs (Delta (B.1.617.2): 570, Alpha (B.1.1.7): 410, Beta (B.1.351): 0, and Gamma (P.1): 0), and the remaining were non-VOCs or those that could not be classified in a single lineage. Monthly monitoring of circulating VOCs from March to September 2021 showed the gradual decrease of the Alpha variant (B.1.1.7) and the dominance of the Delta variant (B.1.617.2) in our study (Figure 4). Furthermore, the circulation trend of VOCs in these regions of the country during this period is relatively similar to the global landscape.^13^ In line with studies of other groups, our study showed that many lineages, especially VOCs, can be recognized successfully using spike sequences.^28^ Still, it should be considered that exact lineage assignment using Spike alone is not possible for many samples whose mutations are not in the Spike gene or whose harboring mutations are compatible with many lineages.^28^ To solve these challenges, recently, O’Toole et al proposed the term “lineage set”, which describes a series of Pango lineages concordant with detected mutations in a given spike gene sequence.^28^ Given that the results of our proposed platform are consistent with the results of other studies, it seems that using this method is a practical and valuable way to identify VOCs in the country.

 In conclusion, our proposed method was practical for rapid and straightforward monitoring of SARS-CoV-2 VOCs for outbreak tracing of SARS-CoV-2 in our country.

## Supplementary Files

Supplementary file 1. List of sample identities, detected mutations, and lineage assignment for 1028 samples between March 2021 and September 2021.

Supplementary file 2. Comparison between mutations and lineages detected by proposed Sanger-based Platform and NGS Platform.

## References

[1] Zhu N, Zhang D, Wang W, Li X, Yang B, Song J (2020). A novel coronavirus from patients with pneumonia in China, 2019. N Engl J Med.

[2] Hu B, Guo H, Zhou P, Shi ZL (2021). Characteristics of SARS-CoV-2 and COVID-19. Nat Rev Microbiol.

[3] WHO Coronavirus (COVID-19) Dashboard. Available from: https://covid19.who.int/.

[4] Lu R, Zhao X, Li J, Niu P, Yang B, Wu H (2020). Genomic characterisation and epidemiology of 2019 novel coronavirus: implications for virus origins and receptor binding. Lancet.

[5] Kadam SB, Sukhramani GS, Bishnoi P, Pable AA, Barvkar VT (2021). SARS-CoV-2, the pandemic coronavirus: molecular and structural insights. J Basic Microbiol.

[6] Severe Acute Respiratory Syndrome Coronavirus 2 Isolate Wuhan-Hu-1, Complete Genome. Available from: https://www.ncbi.nlm.nih.gov/nuccore/1798174254.

[7] V’Kovski P, Kratzel A, Steiner S, Stalder H, Thiel V (2021). Coronavirus biology and replication: implications for SARS-CoV-2. Nat Rev Microbiol.

[8] Tao K, Tzou PL, Nouhin J, Gupta RK, de Oliveira T, Kosakovsky Pond SL (2021). The biological and clinical significance of emerging SARS-CoV-2 variants. Nat Rev Genet.

[9] Daniels RS, Harvey R, Ermetal B, Xiang Z, Galiano M, Adams L (2021). A Sanger sequencing protocol for SARS-CoV-2 S-gene. Influenza Other Respir Viruses.

[10] Harvey WT, Carabelli AM, Jackson B, Gupta RK, Thomson EC, Harrison EM (2021). SARS-CoV-2 variants, spike mutations and immune escape. Nat Rev Microbiol.

[11] Anaclerio F, Ferrante R, Mandatori D, Antonucci I, Capanna M, Damiani V (2021). Different strategies for the identification of SARS-CoV-2 variants in the laboratory practice. Genes (Basel).

[12] Genomic Sequencing of SARS-Cov-2: A Guide to Implementation for Maximum Impact on Public Health. Geneva: WHO; 2021. Available from: https://www.who.int/publications/i/item/9789240018440.

[13] Shu Y, McCauley J (2017). GISAID: Global initiative on sharing all influenza data - from vision to reality. Euro Surveill.

[14] World Health Organization (WHO). Tracking SARS-CoV-2 Variants. Available from: https://www.who.int/en/activities/tracking-SARS-CoV-2-variants/.

[15] World Health Organization (WHO). Guidance for Surveillance of SARS-Cov-2 Variants: Interim Guidance, 9 August 2021. Available from: https://www.who.int/publications/i/item/WHO_2019-nCoV_surveillance_variants.

[16] Han P, Li L, Liu S, Wang Q, Zhang D, Xu Z, et al. Receptor binding and complex structures of human ACE2 to spike RBD from omicron and delta SARS-CoV-2. Cell 2022;185(4):630-40.e10. 10.1016/j.cell.2022.01.001. PMC8733278 35093192

[17] He X, Hong W, Pan X, Lu G, Wei X (2021). SARS-CoV-2 Omicron variant: characteristics and prevention. MedComm (2020).

[18] Fattahi Z, Mohseni M, Jalalvand K, Aghakhani Moghadam F, Ghaziasadi A, Keshavarzi F (2022). SARS-CoV-2 outbreak in Iran: the dynamics of the epidemic and evidence on two independent introductions. Transbound Emerg Dis.

[19] Fattahi Z, Mohseni M, Beheshtian M, Jafarpour A, Jalalvand K, Keshavarzi F (2022). Disease waves of SARS-CoV-2 in Iran closely mirror global pandemic trends. Arch Iran Med.

[20] Bezerra MF, Machado LC, do Carmo Vasconcelos de Carvalho V, Docena C, Brandão-Filho SP, Ayres CFJ (2021). A Sanger-based approach for scaling up screening of SARS-CoV-2 variants of interest and concern. Infect Genet Evol.

[21] Li C, Tian X, Jia X, Wan J, Lu L, Jiang S (2021). The impact of receptor-binding domain natural mutations on antibody recognition of SARS-CoV-2. Signal Transduct Target Ther.

[22] La Rosa G, Mancini P, Bonanno Ferraro G, Veneri C, Iaconelli M, Lucentini L (2021). Rapid screening for SARS-CoV-2 variants of concern in clinical and environmental samples using nested RT-PCR assays targeting key mutations of the spike protein. Water Res.

[23] Paden CR, Tao Y, Queen K, Zhang J, Li Y, Uehara A (2020). Rapid, sensitive, full-genome sequencing of severe acute respiratory syndrome coronavirus 2. Emerg Infect Dis.

[24] Lhomme S, Latour J, Jeanne N, Trémeaux P, Ranger N, Migueres M (2021). Prediction of SARS-CoV-2 variant lineages using the S1-encoding region sequence obtained by PacBio single-molecule real-time sequencing. Viruses.

[25] Hodcroft EB, Zuber M, Nadeau S, Vaughan TG, Crawford KHD, Althaus CL (2021). Spread of a SARS-CoV-2 variant through Europe in the summer of 2020. Nature.

[26] When Do I Use Sanger Sequencing vs NGS? Available from: https://www.thermofisher.com/blog/behindthebench/when-do-i-use-sanger-sequencing-vs-ngs-seq-it-out-7/.

[27] Lee SH (2021). A routine Sanger sequencing target specific mutation assay for SARS-CoV-2 variants of concern and interest. Viruses.

[28] O’Toole Á, Pybus OG, Abram ME, Kelly EJ, Rambaut A (2022). Pango lineage designation and assignment using SARS-CoV-2 spike gene nucleotide sequences. BMC Genomics.

